# The day Norway cried: Proximity and distress in Norwegian citizens following the 22nd July 2011 terrorist attacks in Oslo and on Utøya Island

**DOI:** 10.3402/ejpt.v3i0.19709

**Published:** 2012-12-07

**Authors:** Siri Thoresen, Helene Flood Aakvaag, Tore Wentzel-Larsen, Grete Dyb, Ole Kristian Hjemdal

**Affiliations:** 1 Norwegian Centre for Violence and Traumatic Stress Studies, Oslo, Norway; 2 Centre for Child and Adolescent Mental Health, Eastern and Southern Norway; 3 Institute of Clinical Medicine, Faculty of Medicine, University of Oslo, Norway

**Keywords:** terrorism, PTSD, emotions, safety, epidemiology

## Abstract

**Background:**

Terrorism may create fear and stress reactions not only in the direct victims, but also in the general population.

**Objective:**

This study investigated emotional responses in the Norwegian population following the 22nd July 2011 terrorist attacks. We hypothesized that Oslo residents would report a higher level of fear responses compared with people living outside Oslo and that proximity would be associated with early distress and later post-traumatic stress reactions.

**Method:**

Representative samples were drawn from the Norwegian Population Registry. Telephone interviews were conducted 4–5 months after the attacks. The response rate for the Oslo sample (*N*=465) was 24% of the total sample, and 43% of those who were actually reached by phone and asked to participate. Corresponding figures for the sample living outside Oslo (*N*=716) were 19% and 30%.

**Results:**

Our results show strong immediate emotional responses, particularly sadness and a feeling of unreality, in both samples. Jumpiness and other fear responses were significantly higher among Oslo residents. Current level of risk perception was low 4–5 months after the attacks; however, a significant minority reported to feel less safe than before. Geographical and psychological proximity were associated with early emotional responses. Psychological proximity was significantly associated with post-traumatic stress reactions, while measures of geographical proximity were not. Immediate emotional responses, first-week reactions, and first-week jumpiness were uniquely and significantly associated with post-traumatic stress reactions. Post-traumatic stress reactions were elevated in ethnic minorities.

**Conclusions:**

The terrorist attacks seem to have had a significant effect on the Norwegian population, creating sadness and insecurity, at least in the short term. Proximity to the terrorist attacks was strongly associated with distress in the population, and early distress was strongly related to later post-traumatic stress reactions. Our results indicate that psychological proximity is more strongly associated with post-traumatic stress reactions than geographical proximity, and underline the importance of differentiated measurements of various aspects of early distress.

Terrorism aims to shock the public and to get media attention, thereby achieving political aims (Hoffman, 1999). Thus, the targets of a terrorist go beyond the directly affected victims. This study examines the emotional responses of the general Norwegian population following the terrorist attacks in Oslo and on Utøya Island on 22nd July 2011.

Several terrorist attacks have been followed by studies on the responses of the general population. These include the Oklahoma City Bombing (e.g., Smith, Christiansen, Vincent & Hann, 1999), the 9/11 2001 terrorist attacks in the United States (e.g., Schlenger et al., 2002), the 2004 train attacks in Madrid (e.g., Miguel-Tobal et al., 2006), and the 7th July 2005 London bombings (e.g., Rubin, Brewin, Greenberg, Simpson & Wessely, 2005). These studies have shown that terrorist attacks can influence safety perceptions, well-being, and mental health in the general population. Schuster et al. (2001) reported that almost half of the adult general population throughout the country had one or more substantial symptom of stress in the first days following the September 11 attack. In the months following this attack, a majority reported concern that they or their close ones might become a victim of terrorist attacks, although these perceptions declined over time (Huddy, Feldman, Taber & Lahav, 2005). In the first few weeks after the London bombings on 7th July 2005, one third of the London population reported significant stress reactions, and seven months later, this decreased to 11% (Rubin et al., 2005; Rubin et al., 2007).

Geographical proximity to the events seems to be an important predictor of distress in the population. Schlenger et al. (2002) found a post-traumatic stress disorder (PTSD) prevalence of 11% in New York City, compared with 4% in the general US population. Increased mental health problems following 9/11 were also observed in children and adolescents in New York City, especially in those living close to Ground Zero (Hoven et al., 2005; Neria, DiGrande & Adams, 2011). Similar results were reported following the 2004 Madrid attacks (Miguel-Tobal et al., 2006). Other potential predictors include witnessing horrific events, having panic attacks during events, high levels of exposure to events via television, and having felt that oneself or someone close could be injured or killed (Neria et al., 2011).

Research following the terrorist bombing in Madrid on the 11th March 2004 concluded that the prevalence of PTSD in the general population of Madrid was significantly lower than in New Yorkers living close to the World Trade Centre, while the level of depression was comparable in Madrid and New York, and was assumed to have doubled in both cities in the first few months after the attacks (Miguel-Tobal et al., 2006). The potential impact on the population following terrorist attacks and disasters will likely depend upon the type of event, the type of population, and the kind of changes that people will have to adjust to in its aftermath. For example, the Oklahoma City Bombing resulted in very different mental health problems from Hurricane Katrina (North, 2010).

Research indicates strong immediate responsiveness, which decreases over time, in the general population following terrorist attacks (Silver, Holman, McIntosh, Poulin & Gil-Rivas, 2002). Longer term effects are more uncertain. Rubin et al. (2007) found certain reactions, such as perceived threat (43%) and a more negative world-view (61%), to be maintained at high levels 7 months after the attacks. A significant minority (28%) reported that they had changed their behaviour by reducing certain activities or by being more careful, and 19% had actually travelled less after the London bombings. The most important predictor for psychological reactions and behavioural change after 7 months was the degree of exposure and the interpretation of the event as threatening. Other longer-lasting effects of terrorism might include political and social consequences (e.g., Huddy & Feldman, 2011; Morgan, Wisneski & Skitka, 2011).

On 22nd July 2011, Norway experienced two sequential terrorist attacks. First, a car bomb detonated in the Government Quarter in Oslo, the capital of Norway. Eight people were killed in the explosion, 10 were admitted to hospital with severe injuries, and many more were injured. During the chaotic situation and the rescue operation in Oslo, the assailant moved to Utøya Island, 30 km north of Oslo, which hosted a Norwegian Labour Youth summer camp with 564 participants. In the second attack, the assailant shot those whom he came across on the island; most were adolescents or young adults. The Utøya shooting lasted for approximately 90 min. The terrorist left 69 people dead, and many injured, including 56 who were hospitalised with severe injuries. The days following the attacks were characterized by the clean-up, search, and identification on Utøya Island and in the Government Quarter, and country-wide grieving and protesting expressed by high levels of participation in memorials and other ceremonies.

This paper represents the first inspection of the emotional responses in the Norwegian population following the terrorist attacks of 2011. The particularities of each terrorist attack and the characteristics of the society in which the event unfolds, may influence the nature of the responses. Along a number of potentially important social and cultural dimensions, Norway is a social democratic welfare society with a well-developed and accessible public health system, a relatively homogeneous and stable population, stable political conditions, a generally low level of perceived threat, and a low incidence of violent crime. These characteristics, taken together, might constitute protective factors.

Of the four terrorist attacks that have been followed by studies of the general population, only two occurred in Europe. Thus the current study adds to our knowledge of issues that might be relevant in a European context. In addition, the fact that geographical proximity to the terrorist attack primarily occurred in the capital, while psychological proximity potentially could be present all over the country, gave us an opportunity to investigate the relative importance of the two.

The overall aim of this study was to investigate geographical and psychological proximity, and associations with distress following the terrorist attacks of 22nd July 2011, in the general Norwegian population. We hypothesized that because of the geographical proximity of the bomb-site and the potentially higher probability of future terrorist attacks, the citizens of Oslo would show more fear responses than people living in other parts of Norway. Furthermore, we hypothesized that geographical and psychological proximity would be associated with early distress, and that early distress would be associated with post-traumatic stress reactions (PTSRs) 4–5 months later.

## Methods

### Participants and procedure

Two samples were drawn from the General Population Registry of Norway. The samples were drawn as simple random samples based on gender, age, and area of residence. In this registry, all individuals with a permanent residence in Norway are registered with a unique personal number, which includes their date of birth and sex. The registry also has information about their last known permanent address. The samples were representative of the general population with regard to gender, age, and area of residence within the age range of 16–75 years. One sample constituted a representative sample of the total Norwegian population (*N*=4500), and the other sample constituted a representative sample of residents of Oslo, the capital of Norway, where the bomb exploded (*N*=1500). As the first sample also included residents of Oslo, we report response rates separately for residents of Oslo and residents in areas outside of Oslo, independent of which sample they were originally part of.

All individuals first received a postal invitation letter with information about the study, and they were subsequently phoned and asked to consent to participating. Those who consented were interviewed by telephone 4–5 months after the terrorist attacks. The only exclusion criterion was inability to participate, for example because of language problems, difficulties in hearing, mental incapacity, or intoxication, as evaluated by the interviewer. Telephone interviews were conducted by the market research organization Ipsos MMI 4–5 months after the terrorist attacks. The mean interview time was approximately 30 min.

### Participation and non-participation

Figures for individuals living outside Oslo are given in parentheses. Personal invitation letters were mailed to 2,065 [3,935] individuals. Altogether, 64 [65] letters were returned because of an incorrect or insufficient address, 89 [89] individuals met the exclusion criterion, and 96 [441] individuals did not want to be contacted by telephone, leaving 1,816 [3,340] individuals eligible for telephone interview. A telephone number could not be identified for 348 [661] individuals, leaving 1,468 [2,679] individuals actually called by phone. Of these, 396 [529] did not answer the phone after several calls, technical problems occurred in 76 [149] cases, and other problems occurred in 9 [34] cases, resulting in 987 [1967] individuals successfully contacted by phone and asked to participate. Of these, 524 [1,247] declined to participate, and 463 [720] completed the interview.

The overall response rate was 24.2% (463 participants out of 1912 who presumably received an invitation letter) for the Oslo sample and 19.0% (720 participants out of 3,781 who presumably received an invitation letter) for the “outside Oslo” sample. The response rate for individuals who actually replied to the request for participation was 42.8% for the Oslo sample (463 participated, 524 declined on telephone, and 96 did not want to be contacted by telephone), and 29.9% for the “outside Oslo” sample (720 participated, 1,247 declined on telephone, and 441 did not want to be contacted by telephone). Two individuals were later moved from the “outside Oslo” to the Oslo sample because they self-reported living in Oslo, and two individuals were deleted from the “outside Oslo” sample due to incomplete data, leaving totally 465 respondents in the Oslo sample and 716 respondents in the “outside Oslo” sample.

We investigated potential systematic differences between responders and all non-responders, and between responders and rejecters in both samples, and detected bias related to socio-demographic variables in some analyses (Annex 1). For example, women were over-represented in the Oslo sample, but not in the outside Oslo sample. Responders in both samples were slightly and significantly older than non-responders, but not older than rejecters. Marital status did not differ significantly between responders and non-responders or rejecters in the Oslo sample. However, married individuals were over-represented among responders in the outside Oslo sample, compared to all non-responders. There were no significant differences in county of residence between responders and non-responders or rejecters within the outside Oslo sample.

In addition, we performed analyses within responders of associations between number of calls required to get in touch and socio-demographic variables, proximity, and emotional reactions. These “hard to contact” analyses do not give any support to the hypothesis that individuals with more exposure or stronger reactions were easier to contact. Details are described in Annex 1. Flow charts and further details on non-responder and “hard to contact” analyses can be obtained from the first author upon request.

The Regional Ethical Committee for Medical Research approved this study. Although the risk of harm to the study participants was considered low, the telephone interview included an ethical safeguard. Individuals who confirmed that they were upset at the end of the interview were offered a brief clinical follow-up by an external therapy centre not associated with the study.

## Measures

### Demographics

Gender, age, marital status and geographical region of residence were recorded from the National Population Registry. Ethnicity, religious affiliation, education level, occupational status, and household members were self-reported.

### Geographical proximity to the terrorist attacks

The bomb exploded in the centre of Oslo, the capital of Norway. Being in or near Oslo city was defined as being located within the borders of Oslo municipality or in one of the surrounding municipalities. Being in the centre of Oslo was defined as being located within 500 metres of the main street and the bomb-site.

Sensory impressions were measured by two dichotomous (yes/no) questions that asked the respondents whether or not they had: (1) heard the sound of the explosion; or (2) seen the smoke from the explosion.

### Psychological proximity to the terrorist attacks

Participants were asked a series of dichotomous (yes/no) questions to determine whether they: (1) became worried about the safety of somebody close to them when they got to know about the terrorist attacks; (2) knew someone who was on the Utøya Island at the time of the shooting; (3) knew someone who was at the Government Quarter at the time of the bombing; (4) knew someone who was killed in the terrorist attacks; (5) knew close relatives of the terrorist's victims; or (6) knew a first responder who took part in the disaster response during the first week following the terrorist attacks. If any of these questions were answered affirmatively, the respondents were asked about their relationship with the person whom they knew or worried about (close family, husband/wife/partner/boyfriend/girlfriend, friends or other close relations).

Participation in ceremonies for the terrorist attack victims was measured by a series of dichotomous (yes/no) questions to determine whether the respondent had: (1) lit candles/laid flowers; (2) taken part in a flower march/torch march; (3) taken part in gatherings in churches or mosques; (4) sent/signed condolence protocols; (5) taken part in memorial services; or (6) taken part in other ceremonies or rituals.

Respondents were asked to report on approximately how many hours they had spent following the news: (1) Friday 22nd July from 15:45 h until you went to bed; (2) Saturday 23rd July from when you got up until you went to bed; and (3) Sunday 24th July from when you got up until you went to bed. The reported hours spent following the news each day were summed to a total score to reflect the total hours spent following the news during the weekend after the terrorist attacks.

### Immediate responses, first-week reactions and jumpiness, risk perceptions and PTSRs

Immediate responses: Respondents were asked how much, *during the weekend following the terrorist attacks*, they had responded with: (1) A feeling of unreality; (2) anger or fury; (3) fear; (4) sadness; (5) unrest; and (6) powerlessness, with a four-point Likert-type scale ranging from 0 (not at all) to 3 (extremely). Cronbach's alpha for the six items was 0.77.

First-week reactions: Respondents were asked whether, in the first week following the terrorist attacks, they: (1) had difficulties concentrating on things other than the terrorist attacks; (2) felt sad; (3) had a reduced interest in, or were unable to perform, planned activities; (4) had problems sleeping. Cronbach's alpha for the four items was 0.73. In addition, respondents were asked whether they felt an increased sense of connectedness with other people. Response options for all items were: no; yes, a bit; and yes, a lot. First-week jumpiness: Respondents were asked whether, during the first week following the terrorist attacks, they were more jumpy or on guard than before when: (1) hearing loud sounds; (2) hearing sirens or emergency vehicles; (3) seeing unattended bags or suitcases in public places; and (4) seeing someone looking suspicious. Response options were: no; yes, a bit; and yes, a lot. Cronbach's alpha for the four items was 0.78.

Risk perceptions: Two questions measured current worry about future terrorist attacks: (1) how worried are you about terrorist attacks in Norway in the near future; and (2) how worried are you that future terrorist attacks might hit you, your family or your friends? Both were measured on an 11-point scale from 0 (not worried at all) to 10 (very worried). An additional question asked the respondents to report potential change or stability in their perceived personal safety on a five-point scale, ranging from 1 (much safer) to 5 (much less safe).

Current PTSRs were measured by nine items from the University of California at Los Angeles Post-traumatic Stress Disorder Reaction Index (PTSD-RI) (Steinberg, Brymer, Decker & Pynoos, 2004). Only items from the second part of PTSD-RI were used, to assess PTSRs in the past month. Responses were recorded on a Likert-type scale from 0 (none of the time) to 4 (most of the time). Psychometric properties for the full scale have been documented elsewhere (Steinberg et al., 2004). The nine items were selected on the basis of previous screening following Hurricane Katrina (Kronenberg, et al. 2010). The mean of the nine items correlated highly with the mean of the full scale in a data set with young survivors from Utøya Island (*r*=0.95, *p*<0.001), and with the mean of the full scale in a data set with parents of the survivors from Utøya Island (*r*=0.97, *p*<0.001). Moreover, the same nine items subset derived from the Child PTSD Symptom Scale (CPSS, Foa, Johnson, Feeny & Treadwell, 2001) correlated highly with the CPSS full scale (*r*=0.96, *p*<0.001) in a sample of trauma-exposed children and adolescents attending treatment in Norwegian out-patient clinics (personal communication, Silje M Ormhaug, August 30th, 2012), indicating high agreement between subset and full scale across samples and instruments. Cronbach's alpha for the nine items was 0.66 in the current study. The PTSD-RI was used to study the variation of stress reactions, and was not used as a diagnostic instrument.

### Statistical procedures

Independent samples t-tests (for continuous data) and Chi-square tests (for categorical data) were used to analyse differences between the Oslo sample and the outside Oslo sample, as well as level of emotional reactions in various exposure groups. In univariate analyses, p-values were adjusted for multiple testing within groups of related tests using the Holm procedure. We used hierarchical linear regression analyses to determine associations between potential predictors and PTSRs, entering demographics and proximity to the attacks as variables in Block 1, and adding immediate emotional responses and first week reactions in Block 2. Because of the observed differences between responders and non-responders, we also ran the regression weighted for gender, age, marital status and geographical region. These variables were available for the total sample from the General Population Registry. Specifically, the regression analyses were repeated using inverse probability weights from a logistic regression for response in the total approached sample by gender, age, ethnicity and geographical region, and results from the original and weighted analyses were compared. All analyses were performed using IBM SPSS Statistics, version 19.

## Results

Characteristics of the sample from Oslo and the sample from other parts of Norway are presented in Table 1. Both samples comprised predominantly ethnic Norwegian/Nordic individuals. However, statistically significant differences between the samples were observed on several measures, including marital status and education.

**Table 1:** Characteristics of the samples

Socio-demographic variables	Oslo (*N*=465) % (*n*)/mean (SD)	Other parts of Norway (*N*=716) % (*n*)/mean (SD)	χ^2^, *p* value/*t*-test *p* value
Gender (female)	56.1% (261)	49.0% (351)	χ^2^=5.703, *p*=0.051^a^
Age (range 16–75)	44.3 (15.6)	45.8 (15.6)	*p*=0.102^b^
Ethnicity Nordic (vs Other)	86.6% (402)	92.2% (658)	χ^2^=9.504, *p*=0.008^a^
Education≤12 years (vs >12 years)	31.1% (146)	53.7% (383)	χ^2^=56.242, *p*<0.001^a^
Working/studying (vs unemployed/retired)	83.2 (386)	77.7% (554)	χ^2^=5.268, *p*=0.051^a^
Married or cohabitating (vs unmarried, divorced, separated or widowed)	38.9% (181)	51.9% (371)	χ^2^=19.019, *p*<0.001^a^

a Degrees of freedom=1.

b Degrees of freedom=1,179.

### Geographical and psychological proximity to the terrorist attacks

A large proportion of the respondents from Oslo reported being in or near Oslo at the time of the bombing (55.0%), and/or sensory impressions from the bombing, including hearing the sound of the explosion or seeing the smoke from the bomb-site (34.1%).

In both samples, psychological proximity to the terrorist attacks was prevalent, for example, by worrying about the safety of someone close, or having personal relationships with the victims, survivors, or their relatives (Table 2). Of the 654 individuals who worried about the safety of at least one person, 82.9% (542) worried about close family members, partners/boyfriends/girlfriends or close friends. Among the 300 individuals who reported knowing somebody who was present either on Utøya Island or at the Government Quarter at the time of the terrorist attacks, 51.7% (155) reported the person to be a close family member, partner/boyfriend/girlfriend or close friend. The vast majority of respondents, both in and outside Oslo, had taken part in various ceremonies in honour of the terror victims, such as lighting candles/laying flowers (Oslo: 63.4%, outside Oslo: 50.2%), taking part in flower march or torch march (Oslo: 23.3%, outside Oslo: 20.9%), or memorial services (Oslo: 20.1%, outside Oslo: 18.7%).

**Table 2:** Proximity to the terror and participation in ceremonies in Oslo residents compared with other parts of Norway

*Proximity*	Oslo (*N*=465) % (*n*)	Other parts of Norway (N=716) % (*n*)	χ^2^, *p* value
Worried about the safety of someone close	65.4% (303)	49.2% (351)	χ^2^=29.896, *p*<0.001
Knew someone at the Utøya Island/the Gov. Quarter	28.3% (84)	23.7% (52)	χ^2^=3.065, *p*=0.133
Knew someone who was killed	7.1% (33)	6.7% (48)	χ^2^=0.065, *p*=0.799
Knew next of kin or close friends of victims or survivors	44.9% (207)	42.2% (299)	χ^2^=0.848, *p*=0.714
Participation in ceremonies	82.1% (380)	71.3% (507)	χ^2^=17.594, *p*<0.001

Degrees of freedom=1.

Respondents in both samples reported spending an extensive amount of their time following the news during the weekend 22–24 July 2011, with total means of 17 and 16 h, respectively.

### Immediate responses and first-week reactions to the terrorist attacks

A large proportion of the respondents reported that they cried during the weekend following the terrorist attacks. Crying was reported significantly more often in the Oslo sample (54.5%) than in the rest-of-Norway sample (44.6%; χ^2^=10.838, *p*=0.001).

During the first weekend following the terrorist attacks, a high intensity of affective responses was observed in both groups (Table 3). Strong feelings of fear and anger were observed but were nevertheless the least prevalent emotions in both samples. Sadness and a feeling of unreality were the most predominant responses. Only 3% (3.0% and 2.7%) in each sample reported that they had not reacted with sadness “at all” during the first weekend.

**Table 3:** Immediate emotional responses in Oslo residents compared with other parts of Norway

	Oslo (*N*=465) % (*n*)		Other parts of Norway (*N*=716) % (*n*)		
		
Immediate emotional responses	Not at all/a little	Quite a bit/extremely	Not at all/a little	Quite a bit/extremely	χ^2^, *p*-value^a^
A feeling of unreality	12.9% (59)	87.1% (400)	11.2% (79)	88.8% (628)	χ^2^=15.233, *p*=0.008
Anger or fury	56.6% (259)	43.4% (199)	55.7% (386)	44.3% (307)	χ^2^=1.756, *p*=1.000
Fear	59.9% (276)	40.1% (185)	71.9% (505)	28.1% (197)	χ^2^=24.495, *p*<0.001
Sadness	10.8% (50)	89.2% (412)	13.0% (92)	87.0% (613)	χ^2^=3.122, *p*=1.000
Unrest	38.1% (176)	61.9% (286)	47.3% (331)	52.7% (369)	χ^2^=11.789, *p*=0.020
Powerlessness	32.5% (148)	67.5% (307)	30.8% (211)	69.2% (474)	χ^2^=1.440, *p*=1.000

Degrees of freedom=3.

a χ^2^ values calculated from the full scale (0–3).

In the first week following the terrorist attacks, a majority in both samples felt sad (85.9% and 84.0%), or they found it difficult to think about anything other than the terrorist attacks (61.4% and 56.9%). Less prevalent was sleep problems (19.6% and 13.5%) and a reduced interest in planned activities (45.6% and 37.1%). A substantial minority in both samples reported jumpiness in various situations during the first week following the terrorist attacks. All jumpiness reactions were more prevalent in the Oslo sample (Table 4).

**Table 4:** First-week reactions and jumpiness in Oslo residents compared with other parts of Norway

	Oslo (*N*=465) % (*n*)			Other parts of Norway (*N*=716) % (*n*)			
		
First-week jumpiness	No	Yes, a bit	Yes, a lot	No	Yes, a bit	Yes, a lot	χ^2^, *p* value
At loud sounds	63.8% (293)	18.7% (86)	17.4% (80)	80.5% (568)	11.5% (81)	8.1% (57)	χ^2^=41.335, *p*<0.001
At sirens/emergency vehicles	65.2% (301)	19.9% (92)	14.9% (69)	81.0% (573)	12.9% (91)	6.1% (43)	χ^2^=41.151, *p*<0.001
When seeing unwatched bags or suitcases at public places	76.3% (341)	14.1% (63)	9.6% (43)	88.4% (622)	7.8% (55)	3.8% (27)	χ^2^=30.322, *p*<0.001
When seeing someone looking suspicious	77.4% (357)	14.8% (68)	7.8% (36)	86.0% (606)	9.4% (66)	4.7% (33)	χ^2^=14.101, *p*=0.001

Degrees of freedom=2.

Table 5 displays univariate associations between selected exposure groups and immediate emotional responses, first-week reactions and first-week jumpiness. Both geographical and psychological proximity were significantly associated with immediate responses, first-week reactions and jumpiness.

**Table 5:** Level of immediate reactions, first-week reactions and first-week jumpiness in various exposure groups, independent sample *t*-tests for the differences between exposed and unexposed

		Immediate responses		First-week reactions		First-week jumpiness	
				
Exposure		Mean (SD)	*t*-test *p*	Mean (SD)	*t*-test *p*	Mean (SD)	*t*-test *p*
Geographical proximity							
Living in Oslo	Yes	1.87 (0.62)		0.81 (0.57)		0.42 (0.54)	
	No	1.78 (0.61)	0.038	0.70 (0.51)	0.001	0.22 (0.40)	<0.001
In the centre of Oslo at the time of the explosion	Yes	1.98 (0.57)		0.88 (0.56)		0.60 (0.55)	
	No	1.81 (0.62)	0.053	0.73 (0.53)	0.045	0.28 (0.46)	<0.001
Sensory impressions from the explosion	Yes	1.85 (0.65)		0.83 (0.60)		0.52 (0.56)	
	No	1.81 (0.61)	0.535	0.73 (0.53)	0.045	0.26 (0.45)	<0.001
Psychological proximity							
Worried about the safety of someone close	Yes	1.98 (0.56)		0.86 (0.54)		0.38 (0.52)	
	No	1.62 (0.64)	<0.001	0.59 (0.49)	<0.001	0.20 (0.40)	<0.001
Knew someone at the Utøya Island/the Governmental Quarter	Yes	1.93 (0.60)		0.85 (0.54)		0.34 (0.50)	
	No	1.78 (0.62)	0.001	0.70 (0.53)	<0.001	0.28 (0.47)	0.091
Knew next of kin or close friends of victims or survivors	Yes	1.93 (0.59)		0.84 (0.56)		0.36 (0.51)	
	No	1.73 (0.62)	<0.001	0.67 (0.51)	<0.001	0.25 (0.44)	<0.001

Degrees of freedom=1163–1179.

### Risk perceptions and PTSRs

Respondents did not report a high level of perceived risk for new terrorist attacks. On a scale from 0 to 10, the mean score was 3.5 (SD=2.4) for worry about future terrorist attacks in Norway in the near future, and 3.2 (SD=2.7) for worry that future terrorist attacks might hit the respondent or family members or friends. Risk perceptions were not significantly different between Oslo residents and those living outside Oslo (*t*-test *p*=0.130 and 0.494). However, respondents reported negative changes in overall perceptions of safety: 1.8% (*n*=21) reported feeling safer than before the terrorist attacks, 68.5% (*n*=804) reported no change, and 29.7% (*n*=348) reported feeling less safe than before.

Block 1 in Table 6 displays the results of a multiple regression with PTSRs at 4–5 months as the outcome, by demographic and proximity variables. The general level of reported PTSRs was low, with a mean score of 0.27 (SD=0.34) for people living in Oslo and 0.23 (SD=0.33) for people living outside of Oslo (t-test *p=*0.072). The level of PTSRs was not significantly associated with the time that had passed between the terrorist attacks and the telephone interview (univariate linear regression, B=0.00, 95% CI=0.00–0.00, *p=*0.560). PTSRs were higher in women than in men, higher in individuals with a non-Nordic ethnic background, and increased somewhat with age. Worrying about someone close and having personal relationships with relatives of victims were significantly associated with PTSRs when adjusted for demographics and other exposure variables. In Block 2, immediate and first-week psychological reactions were added. Immediate emotional responses, first-week reactions and first-week jumpiness were highly and significantly associated with later PTSRs. Ethnicity was significantly associated with PTSRs even when adjusted for all the other variables.

**Table 6:** Linear hierarchical multiple regression displaying associations between demographics, proximity and first responses on post-traumatic stress reactions, both samples collapsed. Demographics and proximity variables entered in Block 1, first responses added in Block 2

	Block 1			Block 2		
		
Independent variables	B (95% CI)	Beta	*p* value	B (95% CI)	Beta	*p* value
Demographics						
Gender	0.10 (0.06–0.14)	–	<0.001	−0.02 (−0.06 to 0.01)	–	0.193
Age	0.00 (0.00–0.00)	0.08	0.008	0.00 (0.00–0.00)	0.06	0.032
Ethnicity	0.14 (0.08–0.21)	–	<0.001	0.08 (0.02–0.13)	–	0.009
Geographical proximity						
Living in Oslo	−0.01 (−0.05 to 0.03)	–	0.635	−0.04 (−0.08 to −0.00)	–	0.037
In the centre of Oslo at the time of explosion	0.03 (−0.06 to 0.12)	–	0.504	−0.01 (−0.09 to 0.07)	–	0.822
Sensory impressions from the explosion	0.05 (−0.02 to 0.11)	–	0.169	0.03 (−0.03 to 0.09)	–	0.374
Psychological proximity						
Worried about the safety of someone close	0.09 (0.05–0.13)	–	<0.001	0.03 (−0.01 to 0.07)	–	0.124
Knew someone at Utøya/the Governmental Quarter	0.00 (−0.06 to 0.06)	–	0.993	−0.01 (−0.07 to 0.04)	–	0.636
Knew next of kin or close friends of victims or survivors	0.06 (0.01–0.11)	–	0.014	0.01 (−0.04 to 0.05)	–	0.799
First responses						
Immediate emotional responses	–	–	–	0.07 (0.03–0.10)	0.12	<0.001
First-week reactions	–	–	–	0.19 (0.15–0.23)	0.30	<0.001
First-week jumpiness	–	–	–	0.18 (0.14–0.22)	0.25	<0.001

R2Adjusted: Block 1=0.07, Block 2=0.28. R2 difference test=0.21, *p*<0.001.

Only marginal differences in regression coefficients were observed between the unweighted and weighted regression, with the exception of age. The association between age and PTSRs was somewhat weakened in the weighted regression Block 2 (B=0.001, 95% CI of B=0.000–0.002, β=0.043, *p*=0.104).

## Discussion

Our results show strong immediate responses to the terrorist attacks on 22nd July 2011 in the general population of Norway. Sadness and a feeling of unreality during the first weekend were more prevalent than fear or anger. Sadness and preoccupation with the terrorist attacks were the predominant reactions in the first week after the attacks, while sleep problems were infrequently reported.

Distorted risk perception has been suggested as a potential explanation for PTSRs in the general public, who predominantly has experienced indirect exposure to the event (Marshall et al., 2007). Infrequent, unfamiliar, and very frightening events, such as a terrorist attack, may function as a warning signal of ongoing threat. Perceptions of safety might be altered, and personal risk may be overestimated. In this study, the level of worry about future attacks was low, and so was the reported level of PTSRs. Nevertheless, 30% reported that they felt less safe than they had felt before the attack. Our interpretation of these findings is that the perception of threat in Norwegian society was increased from very low to somewhat higher during the first months following the attacks. Another study, using different measures and samples came to similar conclusions, that Norwegian society was characterized by a somewhat increased fear after 22nd July (Wollebæk, Enjolras, Steen-Johnsen, Ødegård, 2012). However, a small increase in fear in the general population may have important consequences for public health.

Geographical proximity to the terrorist attacks would presumably increase a feeling of personal threat. Our hypothesis that residents of Oslo would report a higher level of fear was supported. Being an Oslo resident was significantly associated with both immediate fear responses and first-week jumpiness, but was not significantly associated with current PTSRs 4–5 months later. These findings are in line with previous research, pointing to geographical proximity as an important predictor of emotional reactions in the public after terrorist attacks (Hoven et al., 2005; Neria et al., 2011; Miguel-Tobal et al., 2006; Schlenger et al., 2002).

Nevertheless, our results seem to indicate that psychological proximity is a more important predictor of PTSRs than geographical proximity. Having a personal connection to relatives of victims, and worrying about the safety of someone close as a result of the terrorist attacks, were uniquely and significantly associated with PTSRs, while the measures of geographical proximity were not. These results underline the importance of psychological closeness and perceived threat to someone close. This is in line with some previous findings, such as those of Neria et al. (2001), who identified perceived threat to oneself or someone close as a predictor of subsequent stress reactions. Few individuals in the general population would presumably perceive an immediate life threat. However, worrying about the safety of somebody close is a reminder of vulnerability, which may be more resistant to habituation than geographical closeness. We would like to add that in addition to perceived threat, psychological proximity by knowing victims or their families might imply being a witness to the suffering and long-term individual consequences of terrorist attacks. Such witnessing may strengthen a variety of negative emotions such as anger, sadness, and hopelessness.

Our findings are consistent with the hypothesis that geographical and psychological proximity to terrorist attacks are associated with early responses, and that these early responses are associated with later PTSRs. Geographical and psychological proximity was not related to PTSRs when adjusted for early responses. Early emotional responses were highly associated with later PTSRs. This finding is consistent with previous studies of the general population after other terrorist attacks (Galea et al., 2002; Tucker, Pfefferbaum, Nixon & Dickson, 2000). In this study, immediate emotional responses, first-week reactions, and first-week jumpiness, were uniquely and significantly associated with PTSRs 4–5 months later. This finding indicates that these three measures tap into separate phenomena, potentially affecting PTSRs in different ways. The immediate emotional responses during the first weekend, dominated by a feeling of unreality and sadness, may primarily reflect cognitive dissonance and immediate empathy with the young victims. First-week reactions, dominated by preoccupation with what happened, continued sadness, and a sense of connectedness to other people, may represent efforts of cognitive integration, support seeking and early coping efforts. Early coping strategies have been associated with later PTSD in a national sample following 9/11 (Silver et al., 2002). The less frequently reported first-week jumpiness is taken as a sign of prolonged alarm response. Our results indicate that differentiated measurement of early responses may be of value for future research.

Several studies of highly exposed groups following the 9/11 attacks have identified ethnic background as a factor associated with subsequent PTSRs (see the review by Neria et al., 2011). We observed that ethnic non-Nordic individuals in our study had an increased risk of PTSRs, even when early distress was adjusted for. This may be related to a perception of ongoing threat specifically against ethnic minorities caused by the terrorist attacks. However, other alternative explanations for this finding, such as socio-economic differences, or previous traumatic experiences not being measured in this study, cannot be ruled out.

### Strengths and limitations

The low response rates in this study may imply biased samples. There has been a substantial decrease in the response rate in population surveys in Norway in the later years, and the rate in this study is not substantially lower than several other studies (Thomsen et al. 2006). The method used in this study, drawing representative samples from the General Population Registry, differs from Random Digit Dialing (RDD) studies with regards to response rates. One advantage with the current study is the potential for thorough investigation of responders and non-responders on registry variables such as gender, age, and marital status. The response rates of 42.8% for Oslo and 29.9% for outside Oslo would be comparable to response rates reported in RDD studies. The potential for bias calls for caution when interpreting the results, especially in relation to the reported prevalences. The associations between variables would presumably suffer less from a potentially biased sample. The weighting of the regression analyses based on gender, age, marital status and geographical region did not indicate that bias affected the estimates significantly. The analyses of the number of calls necessary to make contact did not support the assumption that affected individuals were easier to make contact with, and therefore were over-represented.

Time is essential when investigating PTSRs. In this study, PTSRs were measured 4–5 months after the terrorist attacks and we cannot be sure whether the observed reactions would be similar in the same population in the period before or after our data collection. Our respondents were asked to remember their reactions 4–5 months earlier, and recall that bias may have occurred. Immediate responses and first-week reactions may have been forgotten, or may have been affected by current emotional conditions, and hence, the associations between early responses and later PTSRs may have been overestimated. When comparing Oslo residents with those residing outside Oslo, we cannot rule out the possibility that the observed differences may be explained by factors not measured in this study (such as geographical differences in mental health problems, unemployment, and other risk factors). Furthermore, the cross-sectional nature of this study implies that we must limit our interpretations to associations rather than causal relationships.

Strengths of this study include sufficiently large sample sizes, an extensive interview that included a large number of variables, and a very low number of missing values for individual variables.

We conclude that the Norwegian general population was strongly affected by the terrorist attacks on 22nd July 2011, with sadness and a feeling of unreality much more frequently reported than fear. More fear reactions were observed in Oslo citizens than in people residing in other parts of the country. Although the general perception of risk seemed low, a significant minority reported feeling less safe than before. Immediate responses, first-week reactions, and first-week jumpiness were significantly associated with proximity to the terrorist attacks. Psychological proximity was significantly associated with PTSRs 4–5 months after the attacks, as were immediate responses, first-week reactions and first-week jumpiness. PTSRs were elevated in ethnic minorities.

## Annex 1

**Analyses of potential systematic differences between responders and non-responders, and between responders and rejecters, and analyses of associations between number of calls to get in touch and socio-demographic variables, proximity, and emotional reactions within responders**.

*Analyses of potential systematic differences between responders and non-responders, and between responders and rejecters*.

We investigated potential systematic differences both between responders and all non-responders and between responders and rejecters (those who did not want to be contacted by telephone and those who declined when called). In the Oslo sample, women were over-represented among responders, compared with both all non-responders (56.6% female responders/49.1% female non-responders, χ^2^=7.793, *p*=0.005) and rejecters (47.9% female rejecters, χ^2^=8.004, *p*=0.005). This gender bias was not observed in the “outside Oslo” sample. Responders, in both the Oslo sample and the “outside Oslo” sample, had a higher mean age than non-responders, but not rejecters. The mean age of the Oslo sample was 44.5 (SD=15.5) for responders and 41.2 (SD=14.9) for all non-responders (t-test, *p*<0.001), and the mean age of the “outside Oslo” sample was 45.7 (SD=15.6) for responders and 43.4 (SD=16.3) for all non-responders (t-test, *p*<0.001). Married individuals were over-represented among responders in the “outside Oslo” sample, compared to all non-responders, but not compared with rejecters (51.5% married responders/44.5% married non-responders, χ^2^=11,503, *p*=0.001). Marital status did not differ significantly between responders and non-responders or rejecters in the Oslo sample. With regard to geographical bias, participation in the study was higher in Oslo than in other parts of Norway, as is evident from the response rates described above. There were no significant differences in county of residence between responders and non-responders or rejecters within the “outside Oslo” sample.

*Analyses of associations between number of calls to get in touch and socio-demographic variables, proximity, and emotional reactions within responders*.

However, these analyses of socio-demographic differences between responders and non-responders cannot tell us whether the sample is biased in the variables under investigation. It may be likely that individuals who were especially affected by the terrorist attacks considered the study to be more relevant to them, potentially resulting in an over-representation of affected individuals. We hypothesized that those individuals who were most interested in the study would, after receiving the invitation letter, make themselves more available by telephone, resulting in fewer calls necessary to get in touch, and that those who were “hard to contact” and required many calls would look more like non-responders. Similar procedures have been used previously, as non-responders are believed to behave more like late responders (Danice, Jackson, & White, 2012). Hence, we analysed associations between the number of calls required to make contact and socio-demographic variables, proximity, and emotional reactions within responders. Gender, marital status, education and living in Oslo versus other parts of Norway were not associated with number of calls required. Individuals who were not working or studying, however, needed fewer calls (mean number of calls for those working or studying=5.1 [SD=4.7], vs. those not working or studying=3.9 [SD=4.7], Mann-Whitney U test *p*<0.001). Likewise, significantly fewer calls were necessary for those in age groups between 55 and 70 years of age (mean number of calls=3.80–3.83), than for those in younger age groups (mean number of calls=4.2–6.4; Kruskal–Wallis test, *p*<0.001). Both of these findings may represent increased availability.

Those who were easy to contact and those who were not did not differ significantly in proximity to the terrorist attacks, level of immediate responses to the terrorist attacks, or first-week jumpiness, except that more calls were required to contact those who personally knew the relatives of victims (mean number of calls=5.2 [SD=5.2]) than those who did not (mean number of calls=4.6 [SD=4.4]; Mann-Whitney U test, *p*=0.041), and more calls were required for those who reported jumpiness at loud sounds in the first week (mean number of calls for “a lot”=5.6 [SD=5.7], for “a bit”=5.1 [SD=4.4], and “no”=4.7 [SD=4.6]), than those who did not (Mann-Whitney U test, *p*=0.042). On all other measures, non-significant trends indicated that higher number of calls required to established contact were associated with higher proximity to the terrorist attacks and higher level of emotional reactions. To conclude, these “hard to contact” analyses do not give any support to the hypothesis that individuals with more exposure or stronger reactions were easier to contact.
